# Gender dynamics in seed systems: female makeover or male takeover of specialized sweetpotato seed production, in Lake Zone Tanzania?

**DOI:** 10.1007/s12571-023-01355-7

**Published:** 2023-03-15

**Authors:** Margaret A. McEwan, Moses S. Matui, Sarah Mayanja, Sam Namanda, Kwame Ogero

**Affiliations:** 1 International Potato Center, Regional Office for Sub Saharan Africa, P.O. Box, Nairobi, 25171- 00603 Kenya; 2 Department of Social Sciences, Wageningen University, Wageningen University and Research, Hollandseweg 1, 6706 KN Wageningen, Netherlands; 3 International Potato Center, P.O Box 22274, Kampala, Uganda; 4 International Potato Center, P.O. Box 22274, Kampala, Uganda; 5 International Potato Center, Tanzania c/o Tanzania Agricultural Research Institute (TARI) –Ukiriguru, PO Box 1433, Mwanza, Tanzania

**Keywords:** Seed enterprises, Gender-based roles, Decentralized vine multipliers

## Abstract

Interest is growing for the development of inclusive seed production models. However, there is limited understanding of gender-based roles and constraints and how these might influence gender relations in seed production. Through a case study on sweetpotato seed production in Lake Zone Tanzania, this article examines men’s and women’s roles in seed production with the introduction of specialized seed practices and a commercial orientation. The study uses data from 17 field-based plot observations and eight sex disaggregated focus group discussions (FGDs) with 33 (51% women and 48% men) decentralized vine multipliers (DVMs). Participatory, gender-based analytical tools were used to obtain an in-depth understanding of gender dimensions and implications of new seed production practices, the resources required and access to those resources. Our findings show that men and women have complementary roles in specialized seed production, and that men increased their involvement in production and commercialization, especially when larger monetary inputs and transactions took place. Women gained new tangible (income) and intangible (knowledge) assets, which enhanced their community status. Women’s contributions to household income became more visible. In conclusion male-takeover did not take place. There were changes in the perceptions around sweetpotato production and gender relations. As women’s contributions to household income became more visible, they were able to negotiate with their husbands on access to key resources to maintain this household revenue stream. We discuss how the new knowledge and skills related to seed production enhanced women’s status in the community. These dynamics initiated changes in gender relations and challenged prevailing community perceptions on gender roles.

**Supplementary Information:**

The online version contains supplementary material available at 10.1007/s12571-023-01355-7.

## Introduction

### Background

In the global south achieving seed security so that farmers have timely access to sufficient quantities of affordable quality seed is a fundamental for food and nutrition security (e.g., Bezner Kerr, 2013; Jones et al., 2014; McGuire & Sperling, 2011). However, men and women farmers have different and or joint roles and responsibilities in seed production, maintenance, and selection practices depending on the crop and socio-cultural context, (e.g., Howard-Borjas, 2003; Mudege & Walsh, 2016; Puskur, personal communication). These roles can be influenced by whether the commodity is deemed to be a food security or cash crop (Doss, 2002). Some commentators maintain that crops considered important for food security such as sweetpotato, tend to be the responsibility of women (Adam, 2014; Gundel, 2009). Others such as Orr et al. (2016) argue that: “when they cease to be known as ‘women’s crops’ that automatically belong to women, control becomes a matter for negotiation”. Mudege and Walsh (2016) also comment that increasing commercialization can result in the displacement of women from producing crops that were previously in their domain. Fischer and Qaim (2012) note that in Kenya, women lost access to income from bananas when banana production was “centralized under men’s control”. The increasing appetite by actors in the agricultural and seed system development arena for commercially viable models of seed production, alongside concerns about the marginalization of women farmers, underscores the importance of understanding how the commercialization and specialization of seed production influences gender-based roles and ensuing benefits. Brearley and Kramer (2020) review different approaches for promoting improved varieties and quality seeds in Africa and note that while there are innovative public and private sector models, there is limited gender analysis of the potential harm or benefit of these models on different seed value chain actors. New seed production technologies may be introduced and the tasks and who performs them may change. Moreover, the technologies may require access to new or additional resources. As Mudege and Walsh (2016) note, some seed production technologies (e.g., rapid multiplication technologies used for vegetatively produced crops (VPCs)) are relatively knowledge, capital, and labor intensive, raising questions about who uses and who benefits from them. Doss and Morris (2001) found in Ghana that gendered differences in technology adoption reflect differences in access to the inputs required. Conlago et al. (2011) comment that women may have been empowered as members of the CONPAPA seed producer groups in the Andes but have also been overburdened. Forsythe et al., (2016) working on cassava state that “women can gainfully participate in new commercial cassava opportunities while maintaining, if not increasing, food security. However, these gender effects are highly dependent on gender norms and household relations”. Kramer and Galiè (2020) argue for a better understanding of gender dynamics and gendered opportunities and constraints in seed systems across different commodities. Moreover, gender analysis is needed to ensure that seed system interventions go beyond reaching women, but also to benefit and empower them (Okali, 2012; Puskur et al., 2021).

This paper presents findings from an exploratory study on how men and women addressed gender-based constraints in accessing resources to perform different sweetpotato seed production tasks and provides insights into changing gender relations. We do not investigate the performance of the planting materials being produced and commercialized by the groups and households, nor do we aim to reflect critically on the farmer business model being used to advance seed system development. We seek to contribute to the discussion on what happens when, after project interventions, opportunities arise for commercialization and revenue, if the crop and its seed practices have been managed predominantly by women.

Adapting the Harvard Analytical Framework (March et al., 1999; Overholt et al., 1985), we first focus on the gender-based division of labor for sweetpotato vine multiplication as the visible manifestation of gender-based roles to understand how roles might have changed with the introduction of specialized seed practices. This is an entry point to understand how men and women manage access to the resources required. But as Kabeer (1994) and Doss (2001) have noted, roles are fluid and dynamic. So, we also explore how roles influence gender relations in decision making and negotiation for access to and use of resources needed for seed production, and how women manage practical and strategic needs through individual or group multiplication activities. We also consider whether increases in tangible and intangible assets might improve women’s capacities to meet practical needs related to living conditions such as food security and employment and strategic needs including access to land and freedom from domestic violence (Moser, 1989), and perhaps influence perceptions of women’s empowerment and agency (Kabeer, 1994, 1999). This study contributes to a wider debate on how gender norms, agency and technology uptake interact, and how the introduction of new technologies and institutional arrangements may change gender norms (Badstue et al., 2018a; Petesch et al., 2018a, b), and, in cases of increased commercial importance, may lead to male-take over.

### The context: decentralized sweetpotato vine production in Lake Zone, Tanzania

#### Sweetpotato seed production in the Lake Zone, Tanzania

In Africa, farmers predominantly access seed for non-hybrid crops from their own farms and local markets (McGuire & Sperling, 2016). The characteristics of the planting material (e.g., hybrid true seed, open-pollinated true seed or vegetatively propagated) lead to differences in both seed production and exchange practices. For non-hybrid crops, farmer managed seed production is usually part of the crop production cycle e.g., the bean crop is harvested, after which it is sorted to select seed for future use and stored separately. Private seed companies increasingly engage in seed production and marketing for potato (*solanum tuberosum*) but have had limited involvement in seed production for other VPCs and non-hybrid crops due to, perishability, bulkiness (McEwan, 2016) and perceived low profitability (ISSD Africa, 2017). Public sector and civil society organizations recognize the importance of non-hybrid crops for food security and have made efforts to strengthen those seed systems with community based and decentralized seed production and distribution approaches (Bentley et al., 2018, van Mele & Bentley, 2011). However, interventions tend to overlook both the contribution of different household members to seed production, selection and maintenance practices, and the needs of different types of farmers (e.g., seed producers versus seed users).

In the Lake Zone of Tanzania, women have primary responsibility for sweetpotato seed sourcing and conservation as described by Adam (2014). Sweetpotato has long been referred to as a “woman’s crop”, considered to be food for women and children, and deeply held cultural practices link women—as key purveyors of food within the family—and sweetpotato. This is rooted in the customs of the Sukuma people who are the majority ethnic group in the area. Traditionally, upon marriage, a couple would move to the husband’s parents homestead for an initial period, and the in-laws would allocate the new wife a plot of land to grow sweetpotato. This was a test to see whether the new wife was able to grow sweetpotato to feed her children and herself. While this practice now only exists in remote rural villages, many sayings or proverbs continue to portray persistent attitudes and beliefs about sweetpotato being a “women’s crop”, and ridicule men who grow and eat it. Yet, there are differences in sweetpotato-related beliefs and practices among ethnic groups who have settled in Lake Zone. Moreover, as staple foods and commercial crops – e.g., maize, rice, cassava, banana, and coffee—are affected by changing climate patterns, disease outbreaks and volatile marketing opportunities, perceptions about sweetpotato are also changing and men are increasingly involved in crop and seed production in different ways.

In Tanzania, sweetpotato is most often grown from vegetatively propagated vine cuttings (also referred to as planting material or seed) selected from the standing crop or from roots left in the field after harvest which then sprout with the first rains. The vines are often considered a common good that are shared freely with kin and neighbors. However, purchase is common at the start of the rains and exchange modalities vary depending on the social relationship and geographical distance between the seed producer and user (McEwan et al., 2020). Surveys have reported that farmers consistently state the lack of adequate quantities of planting material at the start of the rains as a constraint (Barker et al., 2009; Gibson et al., 2009; Kapinga et al., 1995; Sindi et al., 2011). Namanda et al. (2011) documented varied practices for conserving and multiplying planting material. But the use of these prevailing conservation and multiplication practices leads to the accumulation of diseases such as sweetpotato virus diseases, and pests such as the sweetpotato weevil (*Cylas puncticollis*). Moreover, as farmers need to wait at least four to six weeks after rains start for roots to sprout new vines, planting is delayed. Both factors contribute to sub optimal yields particularly when unpredictable rainfall patterns are leading to shorter growing seasons. Whereas if farmers plant early, using disease-free seed, they can obtain higher yields, early food, and income.

#### Marando Bora intervention and decentralized vine multipliers

The release of improved sweetpotato varieties (including biofortified orange-fleshed types) has stimulated the design of seed system interventions with specialized seed practices to ensure the timely availability of quality planting material of the improved varieties to contribute to increased food and nutrition security (McEwan et al., 2017). Quality planting material or seed refers to the genetic, physical, physiological purity and health or phytosanitary status. Seed quality can be measured based on tolerance levels set by farmers and seed producers themselves and implemented using internal quality control measures, and, or the standards are established through a formal seed quality assurance system with external seed inspections.

Between 2010–2012 a seed systems intervention was implemented to increase the availability of quality planting material of white-fleshed sweetpotato (WFSP) and orange-fleshed sweetpotato (OFSP) varieties at the start of the rainy season, in Lake Zone, Tanzania. Project implementers identified two WFSP varieties (Polista and Ukerewe) which were released land races and three beta-carotene rich, OFSP released improved varieties (Ejumula, Jewel and Kabode). All varieties underwent clean-up to remove viruses and indexing to confirm health status. The pathogen-tested tissue culture plantlets were micro-propagated, and then hardened and multiplied under field conditions to provide starter stock for the seed system. Subsequent on-farm trials as part of the intervention demonstrated superior performance of the clean virus-tested material OFSP variety Kabode and WFSP variety Polista showing 43.4% and 24.7% yield increase respectively over farmer saved Polista (Namanda et al., 2012). NGO and CBO partners identified and trained farmers to become DVMs i.e., to conserve, multiply and distribute quality planting material at the community level. The DVMs operated as either individual (male or female) or group enterprises. While group membership was dynamic, groups were majority male, majority female or equal male and female membership. Project implementing partners distributed starter material of the five varieties to the DVMs. They trained the DVMs in several new seed production technologies to increase the multiplication rate and to maintain the quality of the planting material. Rapid multiplication technology (RMT) consists of a set of practices to maximize production of vines during the dry season to be ready for farmers to plant for root production at the start of the rains. RMT uses short cuttings which are closely spaced (e.g., 10 cm × 20 cm) in irrigated and fertilized flat seed beds to maximize vine production per unit area. Over four months, 550 cuttings/m^2^ can be harvested using RMT compared to 25 cuttings/m^2^ using conventional farmer practice (Stathers et al., 2018). The phytosanitary quality of the planting material was maintained by ensuring that: the seed multiplication plot was a minimum isolation distance from sweetpotato root production fields; the starter material was disease free and from a known source, and DVMs scouted the plots on a regular basis to identify and rogue out off-types and plants showing disease symptoms. Separate seed beds were used for each variety, labelled with variety name and date of planting. After harvest the cuttings were packed and labelled with contact details of the multiplier, variety name, and number of cuttings.

At the start of the intervention an emphasis on commercialization led to more male farmers being selected for training as DVMs. However, Badstue and Adam (2011) conducted a gender analysis, and raised concerns that women were being excluded from an activity that had hitherto been their responsibility and from which they earned some income. The study recommended that only women should be trained as DVMs. However, implementing partners argued that to target women only to be trained as DVMs would risk alienating men. Therefore, deliberate efforts were made to include more women, by working with existing farmer groups with a high proportion of female members. By the end of the intervention the proportion of women participating in DVM activities had increased from 56 to 67% (McEwan & David, 2012).

## Methods

This case study was part of a wider study conducted in 2017–2018 to follow up DVMs in Lake Zone, Tanzania, five years after a project intervention ended (McEwan et al., 2017; 2020). The broader study used mixed methods with field-based household interviews, plot observations and Focus Group Discussions (FGDs). Eighty-one of the 88 DVMs who had been trained by the project were successfully contacted by cell phone to determine the status of vine multiplication activities, and whether they were continuing to multiply vines for sale (“continuing”); or they had stopped (“non-continuing”). We purposively selected 24 continuing and non-continuing DVMs to illustrate different agro-ecologies, and different types of DVM (i.e., male and female, individual and group-based seed production). The research team conducted semi-structured interviews and plot observations with the DVMs to record the use and perceptions about current seed practices over three rounds of data collection, November–December 2017, April–May 2018, and November–December 2018. The data presented and discussed in this paper were collected from 17 field-based DVM plot observations conducted in November–December 2017 and October–November 2018 and two workshops where in total eight sex disaggregated FGDs were conducted. (Table 1). Of the 17 plots visited, 24% were managed by group DVMs and 76% by individual DVMs. Thirty-three DVMs (51% women and 48% men from unrelated households) attended the two workshops in May 2018 to explore gender dynamics in specialized seed production. There was some overlap in the sample of DVMs visited for plot observations and those invited to the workshops.

**Table 1:** Study composition by type of DVM, Lake Zone Tanzania 2017–2018

**DVM type**	**Study instrument**			
	**Plot observations**		**Individuals participating in FGDs**	
	**n**	**%**	**n**	**%**
Female majority group DVM	2	12	9	27
Male majority group DVM	2	12	7	21
Female individual DVM	9	52	8	24
Male individual DVM	4	24	9	27
**Total**	17	100	33	100*

*Figure rounded up

*DVM* Decentralized vine multiplier, *FGDs* Focus Group Discussions

In each workshop, two topics were explored through participatory exercises with FGDs with seven or eight members each. Each FGD topic session was followed by a plenary discussion to explore the differences in perceptions across men and women. There was also one plenary discussion on some of the implications of operating as an individual or group DVM. Forty-eight percent of the DVMs participating in the FGDs represented groups, the balance operated as individual DVMs. Six percent were in the age group 18–35 years, 85% in the 36–55 years age group and 9% were 56 years or older. 60% of the DVMs participating in the workshops were continuing to sell vines, while 40% had stopped selling.

The first FGD topic was on gender-based constraints and implications of new seed production practices, the resources required and access to those resources. We used gender-based analytical tools (Terrilon et al., 2013; Mayanja et al., 2016) to stimulate the discussion where each sex disaggregated focus group listed the seed production activities and discussed their perception of the level of involvement by men and women, scoring on a scale of one (low involvement) to three (high involvement). Each group then discussed the resources required for each activity and any gender-based constraints to access those resources. In this paper the scores are presented as an average across the two workshops for the male and female FGDs. The final step for the first topic was for each focus group to prioritize their constraints, the cause, consequences, and potential actions to ameliorate the constraint.

In the second FGD topic, research team members applied two participatory tools: the “Ladder of Life” and “Ladder of Power and Freedom” (Petesch, 2018; Petesch & Bullock, 2018; Petesch et al., 2018a, b), with the latter adapted as a “Ladder of Empowerment” for sweetpotato vine multiplication. Facilitators used the two tools in separate male and female FGDs to guide the DVMs to first categorize their communities into four or five steps on the Ladder of Life – with those at the top step being the most well off, and those at the first step being the least well off in the community. The DVMs used the ladder to describe the socio-economic characteristics of households in their communities in relation to participation in sweetpotato vine multiplication, and where vine multipliers are situated on the “Ladder of Life”. The DVMs discussed and then allocated 100 beans to represent the proportion of their community on each step of the ladder, before and after the Marando Bora intervention. The second tool, the “Ladder of Empowerment”, was used by the DVMs to explain how involvement in sweetpotato seed production had impacted on the DVMs’ self-esteem, ability to make decisions and community status. The six steps on the “Ladder of Empowerment” ranged from the first step which signified low self-esteem and very limited involvement in decisions about the vine multiplication enterprise to the sixth step, where they had full empowerment and capacity to make all the decisions. The DVMs voted secretly about their own level of empowerment by writing their score on a ballot paper. The group facilitator then presented the aggregated scores back to the group as the number on each step of the ladder. The groups finally discussed how the commercialization of sweetpotato vine production had changed their capacity to make decisions about their sweetpotato vine enterprise.

All group work and plenary discussions were conducted in Kiswahili, translated, and transcribed into English. FGDs and semi-structured interview data were coded and analyzed using content analysis to identify the emerging themes related to how roles and responsibilities influenced access to and control of resources, benefits in the household and implications for community gender norms. The data analysis and interpretation of findings have benefited from extensive discussions with colleagues in the CGIAR Gender Platform, and this is indicated as personal communications in the text.

## Results and discussion

### Specialized seed production practices and gender roles

Data from household interviews and seed production plot observations were analyzed to verify the specialized seed production practices that were still used. Because plots could be managed by groups or by individuals, results are presented on a plot basis (n = 17).

In plot observations and DVM interviews we noted 10 different specialized seed production practices. Table 2 shows the percentage of DVM plots in which these practices were still used, ranging between 65% of the DVM plots where irrigation was used, to 18% of the DVM plots where vine labels were provided. Overall, practices to increase vine multiplication rates (i.e., irrigation, short cuttings, seed beds, animal manure, close spacing) were used in a higher percentage of DVM plots; and practices to maintain seed quality (e.g., separation of varieties, isolation distance, labelling of plots and vines), were used in a lower percentage of DVM plots. The benefits from using specialized seed production practices include earlier availability of planting material to capture peak prices at the start of the planting season for root production. Use of disease management practices (use of pathogen tested sourced material, roguing, isolation distances, rotation) helps to ensure that the plot will pass the seed inspection standards and DVMs can sell certified seed. As entrepreneurs these DVMs can establish their reputation and build customer trust in the quality of their seed. They are also contributing to their community’s well-being, by making available improved, nutritious sweetpotato varieties.

**Table 2:** Use of 10 different specialized seed production practices in DVM plots, 2017–2018, Lake Zone Tanzania

**Practice**	**% DVM plots**
***Increase multiplication rate***	
Irrigation	65
Short cuttings	59
Seed beds	47
Animal manure	47
Close spacing	41
***Maintain seed quality***	
Separation of varieties	47
Rouging	41
Isolation from other sweetpotato plots	35
Labelled plot	24
Labelling vines	18

Source: Plot observations during field interviews 2017–2018 (n = 17)

*DVM* Decentralized Vine Multiplier

Table 3 provides a breakdown of plot observations by type of DVM management (female or male majority group; female or male individual DVM) and whether at the time of the study they were continuing to multiply vines for sale (70%) or stopped (30%), by the number of practices they continued to use. This appears to indicate that those who were continuing multiplication for sale, also continued to use a higher number of specialized seed practices.

**Table 3:** Number of specialized seed production practices used under different types of DVM plot management and status, 2017–2018, Lake Zone Tanzania

		**No. of specialized practices to increase multiplication rate**			**No. of specialized practices to maintain seed quality**			
**Type of DVM plot management**	**Status**	**0**	**1–3**	**4–5**	**0**	**1–3**	**4–5**	**N**
Female majority group DVM	Continuing to sell	0	0	2	0	2	0	2
	Stopped selling	0	0	0	0	0	0	0
Male majority group DVM	Continuing to sell	0	1	1	0	2	0	2
	Stopped selling	0	0	0	0	0	0	0
Female individual DVM	Continuing to sell	1	2	2	1	3	1	5
	Stopped selling	2	2	0	3	1	0	4
Male individual DVM	Continuing to sell	0	2	1	1	1	1	3
	Stopped selling	0	1	0	0	0	1	1
**Total & % DVMs**		3 (18%)	8 (47%)	6 (35%)	5 (29%)	9 (53%)	3 (18%)	17

Source: Plot observations during field interviews 2017–2018 (n = 17)

*DVM* Decentralized vine multiplier

### Decentralized vine multipliers’ experiences and perceptions of specialized seed production practices

Some of the practices were more difficult to take-up due to the skills or resources required. Thus, our interest to understand if men and women perceived the responsibilities for these practices differently and how they managed to access the resources they needed. In each workshop the sex disaggregated focus groups discussed and scored the level of involvement for each activity. The scores were then aggregated for the two workshops. Table 4 shows male and female focus group perceptions on roles and level of responsibility for specialized seed production.

**Table 4:** Male and female DVM perceptions on the involvement of men and women in specialized sweetpotato seed production, 2017–2018, Lake Zone Tanzania

**Seed production activity**	**Level of involvement** **(1 = low, 3 = high)**			
	**Male focus groups**		**Female focus groups**	
	**M**	**F**	**M**	**F**
**Site selection**	**2.5**	**2**	**3**	**2.5**
Land preparation & digging	2.75	2.75	1	3
*Seed bed preparation*	*2*	*2.5*	*2*	*3*
*Manure application in seed bed preparation & application of top-dressing fertilizer*	*2.3*	*2.6*	*1.3*	*3*
*Vine sourcing, selection, preparation; spacing, planting*	*2*	*2.5*	*-*	*3*
*Watering: seedbed*	*1.5*	*3*	*0.5*	*2.5*
Weeding	2.5	2.5	2	3
Routine monitoring for pest & diseases	2	2	2.5	3
Harvesting	2.5	2.5	0.5	3
Marketing, selling & promotion	3	2	2.75	3
Use of money/income	2	3	3	3

*DVM* Decentralized vine multiplier; 1 = low level of involvement; 3 = high level of involvement

Scores for male and female FGDs were averaged across the two workshops

Bolded activities indicate those where both male and female FGDs were aligned in perceptions that men had higher level of involvement. *Italicized* activities indicate where both male and female FGDs were aligned in perceptions that women had higher level of involvement

Source: Authors’ transcripts from Bunda and Chato district workshops, Lake Zone, Tanzania. May 2018

Both male and female groups agreed that men had greater involvement in site selection. Seed bed preparation, carrying and application of manure, vine sourcing, weeding, and watering were reported to be the roles of women. However, the two groups diverged in their perception of major roles in land preparation and digging, selling vines and the use of the income. The female FGDs argued that women had a higher level of responsibility for weeding, scouting for pests and diseases, marketing, selling and promotion. On the other hand, the male FGDs considered that both men and women had equal involvement in these activities apart from marketing – where they argued that men were more involved; and in the use of money, where they thought that women had more involvement. Table 5 and the following discussion show the gender-based constraints for specific seed production activities.

**Table 5:** Gender based constraints in accessing resources & performing seed production activities, Lake Zone Tanzania

**Seed production activity**	**Resources required**	**Constraints in accessing resources/ performing activity**	
		**Male**	**Female**
Site selection	Money to rent land Time to choose land	Limited cash to rent land Suitable sites are used by horticulture farmers Environmental by-laws	If exchanging land for labour: “the man will leave you to provide labour” “You can work hard to improve the land, but then owner takes it back”
Land preparation & digging	Tools, money to hire oxen & plough	Limited cash to pay for ox-plough, tools Funerals & community obligations	Limited cash to pay for inputs, tools, casual labour With climate change even sites close to water, the source may dry up
Seed bed preparation	Hand hoe, rope measuring tools	Lack of money when needed for vine activities	No constraints
Manure preparation & carrying, top dressing	Money and transport	Money to purchase manure and pay for transport Failure to use correct measurement of top dressing will lead to wilting	Manure is heavy to carry If left too long in the field it may be stolen
Vine sourcing, selection, preparation; spacing, planting	Labour, knives, tape measure	Keenness and know-how Obtaining seed with quality contrary to expectations	If vines infected by pests and diseases will lose an amount You can waste time in coaching an assistant
Watering: seedbed	Watering can, bucket Pump: fuel, transport	High cost of irrigation inputs In prolonged drought, our involvement changes from issuing instructions to searching for water	Lack of money to hire pump We get very tired Water sources dry up An assistant may put too little water
Weeding	Labour, hand hoe/stick	Late weeding will disturb the crop	Limited money to pay for casual labour Time consuming activity
Routine monitoring for pest & diseases	Time, knowledge on pesticide and dosage	Know-how to control pest and diseases Adulterated pesticide Destruction by domestic and wild animals	Unable to differentiate between signs of diseases and lack of nutrients Hard to get advice from extension officers
Harvesting	Knives, rope, sacks, labels	No constraints	It is time consuming
Marketing, selling & promotion	Advertising fliers, sign board, airtime, record books, pen, receipt book	Buyers want free seed Low price for vines, but high production expenses Reliable market/customers for improved sweetpotato varieties (OFSP)	Lack of customers, sustainable market, customers complain about high price of improved varieties Customers have no knowledge of the benefits of OFSP Inability of extension office to visit High charge for seed inspection
Use of money	Money, planning	Limited participation of the family	The husband can use the money for drinking instead to the planned activities at home

Data were combined from the two workshops

Source: Authors’ transcripts from Bunda and Chato district workshops, Lake Zone, Tanzania, May 2018

#### Site selection, land preparation

For many agricultural crops, site identification, clearing of land and digging are often considered typical activities for men (Table 4). Also, in both the men and women focus groups the male involvement in these tasks was scored higher than females. The DVMs emphasized the importance of good site selection to ensure that the soils and land are suitable and that there is access to water for irrigation (Table 5). However, there are differences in perceptions about the constraints to access resources and perform the activity. The female FGDs raised the issue of the time and cash required to identify and negotiate for suitable land and land preparation for vine production (Box 1).

Box 1 Female FGD on resources required for seed production tasks “It may happen that as the mother you do not have the money to hire land, so you have to involve him [the father] so that we both see if the land is fertile or not and that we agree on price together if it is to hire….”. But as another participant in Chato noted: “Sometimes labour may be exchanged for land, but the man will leave you to offer the labour, as there is limited cash to hire land.” It was also noted that with rented land, a multiplier could work to “make the land better and be sacked in only a season because of envy” . In some areas (Musoma) oxen are used in land preparation, but ridges are still prepared with hand tools. A discussion followed on who can hire the oxen, and which crops have priority. One woman explained “it depends on the family arrangement, the man can say we will start with maize followed by sorghum, then rice and so on, but you know the importance of sweetpotato so whenever I have my money, I will look for oxen, and I give money for my land to be ploughed so that I plant my vines, so it depends on the necessity, if I have money than I can hire ox-plough.”

Source: Female FGDs in Bunda and Chato, May 2018.

#### Seed bed preparation, seed sourcing, selection, preparation, spacing

Both male and female FGDs regarded the preparation of seed beds, seed sourcing and selection as activities with higher female involvement (Table 4). The women reported no constraints in accessing the knife, ruler, bags, rope, and labels required or in performing the activity. However, they noted that if there are pest and diseases in the planting materials, they will have less seed than expected (Table 5). Men noted the constraints that they face are “keenness and know-how” and that sometimes the seeds they obtained were contrary to their expectations. Men have different views on male and female keenness on the measurements for planting (Box 2).

Box 2 Male FGD on resources required for seed production tasks The male FGD are very concerned about the step-by-step requirements for measuring the seed bed and spacing. In Bunda, the men said that measuring is their work: “women say that this is our (men’s) work and that they (women) are unable.” Another male participant added “they are not keen enough with measurements.” However, another male participant interjected: “but there are women who are very swift and good in planting.”

Source: Male FGDs in Bunda and Chato, May 2018.

#### Agronomic practices

Farmyard manure is applied during seed bed preparation and inorganic fertilizer may be used for top-dressing after the first vine harvest. These activities were undertaken jointly, but both sexes agreed that women had more responsibility and involvement (Table 4). The female FGDs noted that limited money is a constraint to buy animal manure, transport it and pay casual labor; also, that the manure is heavy to carry and can even be stolen from the field if it is not spread quickly (Table 5). Manure collection, transport, and spreading are predominantly done by family members – except for better off households who may hire labor. Youth were more involved in manure spreading (together with watering), being relatively unskilled, but labor-intensive activities. Watering – during seed bed establishment and routine watering was seen by both male and female FGDs as a predominantly female activity with assistance from youth and children. The women mentioned, that even with assistants, close monitoring was needed as *“someone else can put a small amount of water and cause a problem”* (Table 5). Female FGD respondents explained that watering is tiring and mentioned lack of money to buy an engine to pump water. Women mentioned that water sources are drying up due to changing rainfall patterns. Men also discussed this, explaining that in drought or dry periods, where water availability or access is a problem, they (men) get more involved. The men mentioned their responsibility to purchase a pump, and additional pipes (e.g., if a well had to be deepened, or a more distant source of water was used); and highlighted that this often involved *“taking funds from one pocket to another”* to support the vine multiplication. The men noted that their responsibility shifts from issuing *“instructions to water”* to having to help search for water themselves (Table 5); in fact, this turns out to be a priority constraint which men identify (Table 6). Both men and women were responsible for weeding. The female FGDs stated that this task was tiring but money was a constraint to hire casual labor. The male FGDs noted that weeding practices differed depending on planting method i.e., using RMT in a seed bed with close spacing, it was difficult to use a *jembe* (hand hoe), so a stick or hands were used, whereas on ridges weeding could be done by *jembe*. The frequency of weeding also depended on the soil type and the extent of irrigation use – as weeds like vines multiply more quickly with additional water (and fertilizer) application. Women noted that they removed weeds while they watered the seed bed or when scouting for pests and diseases. The male FGDs considered that men and women were equally involved in pest and disease management; however, the female FGDs rated their own involvement higher than men (Table 4). There was extensive discussion in both male and female FGDs around this to differentiate between routine monitoring and external inspection (by the regulatory body). Women noted that they carry out routine monitoring at the same time as weeding or watering, or when they check to see if newly planted cuttings have sprouted. In these cases, women considered they were more involved: *“we go to inspect frequently; when I see that there is a disease in the vines, I am uprooting it and taking away so here I also prevent disease by doing this”* (woman, plenary discussion, Bunda)*.* External inspections were carried out by the district crop officer delegated by the Tanzanian Official Seed Certification Institute (TOSCI). One woman explained: *“after inspecting if there is any problem, I will involve the extension officer for the preventing of the diseases”* (woman, plenary discussion, Bunda)*.* The women shared that they were challenged because they were unable to differentiate between symptoms of diseases and signs of nutrient deficiencies and that it was hard to get advice from extension officers, who themselves had transport constraints to get to the field (Table 5). The female FGDs, but not the male FGDs, discussed the high costs for the TOSCI inspections (Table 7) e.g., payment for registration as a seed producer and seed inspections, explaining that the two visits cost TZS 100,000, (approximately USD 43) each.

**Table 6:** Men’s priority constraints for sweetpotato seed enterprises in Lake Zone, Tanzania

**Priority constraints: men**	**Cause**	**Consequence**	**Potential Actions**
Men don’t have **sufficient time** to participate fully in vine activities (C)	Men have a lot of responsibilities as head planners in the family	The involvement of women in vine multiplication increases	Men to participate fully in vine multiplication
Vine activities requires **additional labour** from men and increased resources (C)	Mainly during drought and flooding	The use of household resources from one pocket in to the other (compromising the income status of the family)	We should be keen in the selection of suitable land
Men have the know-how step by step (systematic) but the problem is on **how to delegate this know-how** especially to their spouse (C)	Vine multiplication requires supervision by men	Activities that were delegated to others end up being supervised or done by the men	Men to demonstrate step by step those activities that they wish to delegate to their spouse
**Floods and prolonged droughts** (B)	Weather variability; environmental destruction	Reduced production. Water sources dry up	Follow advice from weather experts; Conservation of the environment
**Lack of reliable market** (B)	Short window of opportunity to sell vines	Vines lost or wasted	Continued engagement and training of community on benefits of using improved seed vines

Source: Authors’ transcripts from Bunda (B) and Chato (C) district workshops, Lake Zone, Tanzania, May 2018

**Table 7:** Women’s priority constraints for sweetpotato seed enterprises in Lake Zone, Tanzania

**Priority constraints: Women**	**Cause**	**Consequence**	**Potential Actions**
**Lack of reliable market for vines and roots** for both OFSP and non OFSP (C&B)	This season over supply in market because of good rains People have no money to buy vines or roots. People don’t know the benefits of OFSP. Farmers have no access to market	Despair to continue production of sweetpotato vines; Varieties will be lost Food insecurity, hunger, malnutrition	DVMs continue to educate the community about OFSP; Learn value addition; Sensitize schools; Have a union- communicate to market products together; Political support to encourage all families to plant sweetpotato for food and business
**TOSCI registration** We need to meet the requirements of good quality seed production e.g., isolation (C)	Difficult to meet requirements for isolation & registration; can’t transport vines to a distant market without label	Difficult to find new markets outside the area. Paying additional money for TOSCI inspection but farmers not willing to pay higher price. Cannot afford cost of registration so decide to multiply only for own use not for sale	It is a government role
**Transport to get roots to market,** show grounds for varieties and advertise vines (C)	DVMs can’t afford own transport to market	Traders coming to buy at a very low price so low income from sweetpotato roots	Government policy for extension officers to reach farmers should be given priority
Lack of **irrigation resources** (pump), and unreliable water sources (B)	Lack of money Change in climate conditions	Multiplication plots dry up. Lack of food	Government should support irrigation infrastructure in the villages, e.g., wells
Lack of **processing machines for sweetpotato products/value addition** (B)	Lack of education about diversified uses of sweetpotato	Limited customers for vines, farmer groups disband, lack of income, lack of nutrition	Promotion of more diversified uses for sweetpotato will encourage customers to buy more vines

Source: Authors’ transcripts from Bunda (B) and Chato (C) district workshops, Lake Zone, Tanzania, May 2018

#### Harvesting and marketing vines

The male FGDs, went into some detail to explain the harvesting, conditioning of seed and preparation for sale, and did not separate these steps from marketing activities in general (Table 5). The female FGDs explained that money is required for the signboard, fliers, and airtime. Women had constraints to access these resources, due to lack of customers. The women noted that customers had no knowledge of the benefits of the improved varieties or complained about the high price of vines (Table 5). The women explained that the extension officer who helps to link them to markets cannot visit them due to lack of funds to purchase fuel. But they also highlighted that their husbands had better market access as they can *“go by bicycle to the nearby village to tell people about the vines”* (woman, FGD, Chato)*.* This highlights women’s limited mobility as a bottleneck to better market access. The male FGDs noted that the buyers (customers) wanted free seed, or offered low prices for vines, yet the DVMs faced high production expenses. They also noted that they were yet to find reliable customers for the nutritious (“*lishe*”) OFSP varieties. Women shared that sometimes customers phoned to tell them to prepare a certain number of bundles of vines; but then *“you wait for him on the agreed day, but he does not show up, first day, second day, third day”; or the customer may say just give me the vines I will pay your money on this day; but not pay as agreed”* (woman FGD, Chato)*.* Female FGDs indicated a higher score for children’s participation in the vine multiplication activities than the male FGDs (Table 4). This may reflect that women encouraged children to work more closely with them so that they were more aware of the different tasks that children were involved in; or that the “ownership” of the crop continued in the woman’s domain. Although it was recognized that children were attending school, they could still help at the weekends. The conversations in the FGDs showed that intergenerational transfer of skills followed gendered patterns, i.e., young girls learned from their mothers and boys followed their fathers’ behavior. The DVMs did not mention isolation distances as a challenge. This may be due to a lack of awareness and limited implementation of the seed regulations. The reasons given for not using seed beds related to the aim to produce both roots and vines to spread risk since the market for vines was unreliable. The DVMs also commented that labelling was not necessary as customers know their preferred varieties. Labeling was used for specific customers i.e., institutional customers, such as NGOs or government extension departments.

#### Priority constraints: a gender perspective

In Table 6 and Table 7 we present gender differences in the prioritization of key constraints in sweetpotato seed production, show how the separate male and female FGDs debated the causes and consequences of these constraints and explore how these relate to practical and strategic needs of men and women. Men prioritized constraints which related to how they planned and supervised the sweetpotato multiplication activities within the household. Women noted practical challenges around transporting roots to markets, which made them more dependent on the local traders who come to the village but who offered lower prices. Women noted other practical constraints around time, cash to hire casual labour for burdensome tasks, and mobility to access more distant markets. But they also argued for their more strategic needs: government policy support for the crop, a value chain approach for sub sector development, effective extension services, market linkages and action to address the effects of climate change.

Both women and men identified the lack of a reliable market for sweetpotato vines as a key constraint. This is due to fluctuating seasonal demand (i.e., in seasons with good rainfall, there are plentiful vines, so farmers do not need to buy them). Also, there is a short window at the start of the rains to sell vines at a premium price. Moreover, women considered the lack of awareness about the nutritional benefits of the OFSP varieties to be a constraint. Women argued that promotion of the diversified uses for sweetpotato roots and better market linkages would increase demand for roots and subsequently customers for their vines. Women also highlighted the importance of getting their seed inspected and labelled so the seed could pass through district border points and so that they could extend their market beyond their immediate area. Both men and women highlighted the role of adverse weather events such as flooding and drought in affecting their vine production activities. They explained that cutting of trees for firewood and charcoal making contributed to flooding and changing rainfall patters and that the drying up of water sources was linked to drought.

#### Operating as individual or group DVMs

The DVMs had a discussion on the advantages and disadvantages of working as an individual or group DVM (Table 8). This highlights the importance of group dynamics in the success or failure of a group. Moreover, women and youth may benefit from being part of a group in being able to pool resources to rent suitable land for vine multiplication and accessing training and inter-generational transfer of knowledge.

**Table 8:** Advantages and disadvantages or working as an individual or group DVM, Lake Zone Tanzania

	**Individual DVM**	**Group DVM**
**Advantages**	*“You will do the work as if it is your only responsibility and employment… to reach your own set goals at your own time”.* *“You will do the work without depending on people’s ideas, apart from the experts”.* *“The person cannot give up even if that person gets problems… s/he has made the production of vines his/her own employment and responsibility”.*	*“The government can easily help groups, as they are able to reach many people at once”* *“The production rises when having good people”.* *“Then there is exchange of experiences….. when we are together, we learn from each other”.* *“When you involve a woman in a good group… women sacrifice a lot with all of their strength and mind and heart”.* *“If women and youth are involved, they participate to get experience which is being taught with the elders”.*
**Disadvantages**	*“The production can slow down when you get an emergency e.g., a funeral… your vines may not be watered and will be dead”.* *“You will not be reached with the services planned by the government”.* *“For a woman she gets problems in individual production because of not getting enough capital which is needed in renting the shamba, but if they are many, they can contribute with what they have to reach the amount needed.*	*“When group work is organized, some people don’t show up and have excuses such as attending funerals or emergencies elsewhere”.* *“There are different kinds of people and behaviors in groups”.* *“This leads to low production because of lazy behavior”.* *“Some people can plan with other thieves to steal vines”.* *“If women start back-biting behaviors this can lead to the falling or death of the group completely”.*

Source: Authors’ transcripts from Bunda district workshop, Lake Zone, Tanzania, May 2018

Key: DVM = Decentralized vine multiplier

### Characterization of sweetpotato vine multipliers and changing perceptions

Table 9 shows the results from the two workshops where male and female FGDs used the “Ladder of Life” tool to describe the socio-economic status of households in their communities and which types of households were involved in sweetpotato vine multiplication. The “Ladder of Life” tool considered five categories of households, which participants described using local indicators. The households on the top and bottom steps were not involved in sweetpotato vine production for sale. Those at the top, the FG participants said, do not multiply vines, but buy vines and cultivate local sweetpotato varieties. Households on the bottom step do not multiply vines, because they have no land to grow crops. The focus groups described how sweetpotato seed producers on the second, third and fourth steps were different. Households on the fourth step had more suitable land for vine multiplication and used water pumps rather than buckets to irrigate, in contrast to those on the second step from the bottom. The DVMs noted that multipliers on the fourth step were unlikely to be members of groups, and that they could afford to hire casual labour. Those on the second step were keen to try out improved technologies, but still operated on a small scale because they relied on family labour. Multipliers on the third step sold planting material of local and improved varieties, but those on the second step were more likely to be multiplying local varieties only. The male focus group in Bunda shared the changes they had observed: *“6–7 years ago the sweetpotato crop was divorced and left to women. Men never involved themselves in the crop. Now it is different with education and seeing the results, you see the difference”. “Before [the Marando Bora project], people used to do horticulture in the lowland, from which they were getting income. “Lishe” (OFSP) was different in that it diversified their income.” “Sweetpotato is not as involving and demanding as horticulture production and is cost effective.”* The male DVMs explained: *“After the Marando Bora project, the households of some participants started jumping from one step to another”. “Many jumped upwards”.*

**Table 9:** Characterization of sweetpotato vine multipliers in the “Ladder of Life”, Lake Zone Tanzania

**Ladder of Life: step 5 (Bunda male FG only)** • They have many cows • They own large chunk of land • They invest in different projects • Their household is educated • **They are not involved in vine multiplication, but buy vines**
**Ladder of Life: step 4** • They have big clean land (five acres and above) and they can cultivate them (FB, MC) • Good and suitable land for multiplication (MC) • They don’t multiply vines, they buy vines, they cultivate local varieties (FB) • They have means of transport (motorbike or vehicle) (MB) • They have large plots of land watering, pumps. They also multiply local varieties (FC) • **They would not like to be in groups;** it will be a waste of their time and they can afford casual labour. It could be that they started in a group, but later outgrew the importance of being in a group (MB)
**Ladder of Life: step 3** • They have average to **big land (10 acres/4 ha) suitable for multiplication** (FB) • **Modern equipment i.e., water pump, oxen** (MB) • They multiply local and improved varieties; they conserve MB varieties in seed beds (FB, MB, FC) • **They were likely to separate vine production in beds; and have a good multiplication plan** (MC) • They will have resources to improve fertility of their land: gather and spread manure (MB)
**Ladder of Life: step 2** • 1–3 acres (0.4 -1.2 ha) intercropped with other crops • They multiply vines (local varieties) in small areas and don’t sell vines, those who are near the water source can conserve, they are ready to study they accept technology easily. (FC) They beg for MB vines (FB, FC) • **They were likely to separate vine production in beds; and have a good multiplication plan** (MC) • They will start small because they are using family labour (MB)
**Ladder of Life: step 1** • They barely afford one meal in a day (MB) • The end of the household is like a drunkard (MB) • There shelter is not permanent (grass thatched) (FB, MB) • They rely on daily wage labour –for their daily bread (MB) • They have older age, or don’t have ability to work (FB) • Children don’t go to school – they help parents/grandparents (MB) • They rely on hand-outs (MB) • They multiply in small plots and mix varieties per bed (MC, FC) • They produce for own use and do not appear to be consistent in what they do (MC) • These **don’t multiply vines**, they don’t have land (FB, MB)

Source: Authors’ transcripts from Bunda and Chato district workshops, Lake Zone, Tanzania, May 2018

*MB* male FGD, Bunda, *FB* female FGD, Bunda, *MC* male FGD, Chato, *FC* female FGD, Chato

#### Changes in capacity to make decisions

After completing the participatory exercise for the “Ladder of Life”, the male and female FGDs used the second tool, the “Ladder of Empowerment”, to discuss their capacity to make decisions about their sweetpotato vine enterprise. Both male and female FGDs showed how their decision-making capacity had changed before and after the intervention on a scale of six. Some men noted that previously women had control over the crop and men were hardly involved in vine production, and so they scored themselves between one and three; but now it was a business which men engaged in to increase their income *“we also have increased knowledge as we continued involving ourselves in the business”* (male FGD, Chato). When scoring their level of decision making in the seed enterprise comparing before and after the project e.g., about allocation of land, choice of varieties to multiply and how to use the money obtained from selling vines, the men debated on their scores between four and six and did not unanimously give themselves the highest score of six. As they explained: *“because when you enforce power, without cooperating with others, you will not bring development in the family”* (male FGD, Bunda). Women also scored themselves between one and three, and between four and six before and after the project respectively. They noted that *“before the project men did not have knowledge ……, after the Marando Bora commercialization, because we were selling, we showed them what we were getting so they start realizing the benefit to the households”* (female FGD, Bunda).

### Influence on household and community practices

The sex disaggregated FGDs provided insights into some of the gender related consequences when sweetpotato vine production became a more specialized activity and how men and women negotiated and managed tasks. The findings showed that with specialization and commercialization there were new or additional tasks for which resources were required: site selection, sourcing starter seed, irrigation, weeding, pest and disease management and marketing. But the FGDs also showed that both women and men considered that there is now more negotiation around the existing roles and decision-making related to their sweetpotato enterprise. Men joined their wives in their vine multiplication activities, ensuring that measurements for seed beds, plant spacing, and fertilizer application were done correctly, and men provided financial or practical support for irrigation during dry spells. Men explained that they delegated tasks and issued instructions. However, if household resources were required, they were more actively involved (e.g., deepening wells, buying a pump). Men said they were preoccupied with using their new knowledge and skills to get measurements correct and their discussion centered around the difficulties of delegating certain tasks that required specific steps and measurements, such as plant spacing, or the correct dose of inorganic fertilizer. Men ranked the problem of delegating tasks and passing on knowledge to the women among their top three priority constraints. In contrast to the men, the women DVMs reiterated how they ensured that knowledge is shared between wife and husband: *“when you go back you have to be open to the father so that he knows what is going on. E.g., with a cassava seminar, I told him everything from the benefits of selling the seed. When I got another seminar, he did not hesitate to allow me and assists because he had the knowledge [as well] and saw the benefits”* (female FGD, Chato).

Previously, income from the sale of vines was received and managed by women. This was often referred to as “money for soap”, i.e., small amounts under the control of women. Access to this kind of income is important as the wife does not need to keep asking her husband for money for items like salt, sugar, and soap. The women noted that *“the man has reduced violence because he is educated now”* (female FGD, Bunda). In Kenya, female control over small amounts of income is also noted to reduce tensions between spouses and potentially domestic abuse (Esther Njuguna-Mungai, personal communication). The husband may be unaware of each of these exchanges of small volumes of vines and money between women, or dismisses them as insignificant e.g., during a field interview with a male DVM, an elderly woman passed by the compound to collect a handful of vines and offered money. The man declined, reflecting that this was just “soap money”. However, in an example from Ukerewe, the husband explained that during a project or when there is an institutional customer who wants to purchase a larger quantity, he manages the transaction and income, but when the main customers were neighbors and farmers in the same village his wife is responsible. This shows that the management of the vine sales also depends on the volume of sales and type of customer.

Women also explained that through commercialization and the increase in income from vine multiplication, men started to realize the benefits to the household. *“When the husband saw that fellow men are busy with sweetpotato vines, he became interested, so he listened to me. And also, because from the selling of vines I bought chickens, I sold them and bought a goat which I still have”* (female FGD, Bunda). Women were keen to ensure harmony and peace in the family, so emphasized the importance of sharing information. They talked about planning the household budget together with their husbands, but they also shared the experience that even with a plan in place, if the husband was the one receiving the cash, he could divert it to other uses, such as “drinking”. Men in the FGDs also reflected on decision making around the use of money from vine sales. Some emphasized the community norm that *“the man decides how it is used”-* but others disagreed, stating that *“we involve each other in such decisions”* (male FGD Chato).

Box 3 shows how women shared the knowledge and skills from the training they received both with their husbands and helped the information spread in the community at large. The community gave greater recognition to women DVMs when they saw the benefits of the new varieties (especially, the nutritious, orange-fleshed varieties), which in turn gave the women greater voice within the household.

Box 3 Women’s view on changes in their status in the community Women in the FGD said that “Those that received training, they spread it to the community and the community changed….” …..and ..... “when people had seen the Marando Bora varieties they started to produce them and sell. The community copied the knowledge…. And men saw the benefits and started to listen to us”. They consider that “it is the result of you contributing to the family when the need is there. We have been seen and we have been teaching the community, even the husbands listen and take advices from their wives and so the change. When we become educated that’s when the families thrive because of the power of women, we will be heard by men because we will have money” (Female FGD, Chato District).

The perceptions around sweetpotato have also changed. Previously men had been ridiculed: “*you are talking about sweetpotato, but you (men) don’t know – you plant the vines upside down”* (female, DVM interview Ukerewe). However, as sweetpotato is increasingly considered a commercial crop, men were now involved in all related production activities. *“Before, sweetpotato was a woman’s crop but of late men are involved in the crop—all activities—because it is now a commercial crop”.* The workshops and FGDs also stimulated reflection by the participants. One male DVM explained during the workshop in Chato: *“in 2012, when I was multiplying and disseminating vines, people wondered what kind of man I was, selling vines. But from this workshop I have been encouraged to go tell other men that sweetpotato is a crop just like any other”.*

## Conclusions

We show the roles and level of involvement of men and women for different tasks in the specialization and commercialization of sweetpotato seed production in Lake Zone Tanzania. Those DVMs who were continuing vine multiplication for sale, also continued to use a higher number of specialized seed practices. Practices to increase vine multiplication rates (i.e., irrigation, short cuttings, seed beds, animal manure, close spacing) were used in a higher percentage of DVM plots; but practices to maintain seed quality (e.g., separation of varieties, isolation distance, labelling of plots and vines), were used in a lower percentage of DVM plots. Some of these practices are more difficult to take-up due to the skills or resources required. Men highlighted constraints which related to how they planned and supervised the sweetpotato multiplication activities within the household. Women noted practical challenges around transporting product to markets, which made them more dependent on the local traders who come to the village but who offered lower prices. Women commented on other practical constraints around time, cash to hire casual labour for burdensome tasks, and mobility to access more distant markets. But women also argued for their more strategic needs: policy support for the crop, a value chain approach for sub sector development, effective extension services, market linkages and action to address effects of climate change.

While men and women engaged in sweetpotato vine multiplication had different strategies for addressing constraints, the overall impression that emerged from our interactions was a combination of complementarity and negotiation of roles in the face of new opportunities offered by the specialization in seed production. The men joined their wives in the identification of suitable land and took on the negotiations to rent the land. Men also gave examples of how they paid additional funds when inputs were needed, for example for irrigation. Women noted that working together within the household they could overcome resource constraints. Men accepted that sweetpotato vine production by the women contributed to household income. Sale of sweetpotato vines continued as a source of income for women, but men acknowledged that this was more than “soap money” and was a tangible contribution to the household budget. There is anecdotal evidence that the husband managed larger transactions, but this may reflect biases in how institutional customers (NGOs and government departments) operate. Or we may too readily interpret the increasing involvement of the men with larger transactions and market opportunities as “taking” income opportunities away from the women. Our experiences rather show that the man might take the role of managing the larger transactions because that is a traditional role which women are not yet ready to assume. The commercialization lifts up the entire household, which is recognized by both men and women. These findings echo Badstue et al. (2018b) who argued that married women are better able to innovate when they have their husbands’ acceptance and support. It also points to complementary roles for men and women rather than competition for the income opportunities.

Women also brought intangible assets such as technical knowledge (about seed bed preparation, planting methods, pest and disease management), negotiation skills and self-esteem to the joint discussions on decisions around the seed enterprise and how subsequent income should be used for the household benefit. Community members recognized the knowledge and skills of women seed producers, and the benefits from access to the improved nutritious varieties. Women spoke of their enhanced self-esteem and community status, and this contributed to changes in gender perceptions and intra-household gender negotiations. While this underlines how gender relations are dynamic, the process is context specific and broader development processes and other types of social relations also play a role. As Quisumbing and Pandolfelli (2010) note, gender norms do not change overnight, and it is important to balance women’s short-term practical needs with longer term strategic issues around changing norms.

It was also noted by the DVMs that operating as a group can also contribute to addressing some of the practical and strategic constraints that women and youth face in seed production. While group dynamics can determine the success or failure of a group, women and youth may benefit from being part of a group in being able to pool resources to rent suitable land for vine multiplication and accessing training and inter-generational transfer of knowledge. Moreover, a narrow focus on differences between men and women can mask more important differences among women, such as age; marital status; education level; and size of landholding, or socio-economic status (Quisumbing & Pandolfelli, 2010). The discussions highlighted that household access to land and the socio-economic status of the household may be more relevant to the success of the sweetpotato seed enterprise than gender norms and associated practices.

The insights from this study made us question the way the “take-over” of crop production by men is usually portrayed in literature. The DVMs thought that sweetpotato vine multiplication had bought them knowledge and income benefits that reflected as changes in household socio-economic status, and not in differences between men and women. There may be trade-offs in challenging gender norms in order to address strategic needs and every practical development intervention impacts on power relations. There can also be risks of male take-over of responsibilities and benefits from seed production. In some contexts, solely female run seed entrepreneurship may be more appropriate (Puskur, personal communication). However, there are others where co-ownership and shared responsibilities in seed enterprises by men and women may lead to more sustainable seed enterprises and be a much more effective vehicle for changes in gender relations. We need to acknowledge that not all households are the same and change comes about in different ways and speed.

## Supplementary Information

Below is the link to the electronic supplementary material.

Supplementary file1 (DOCX 16 KB)

## Data Availability

Data that is not presented in the article is available on request.
