# Supplementary material for: Gender dynamics in seed systems: female makeover or male takeover of specialized sweetpotato seed production, in Lake Zone Tanzania?
Source: Food Secur. 2023 Mar 15;15(3):693–710. doi: 10.1007/s12571-023-01355-7 (PMC10202969; doi:10.1007/s12571-023-01355-7)
Supplement: Supplementary file 1 — Supplementary file1 (DOCX 16 KB) [file 12571_2023_1355_MOESM1_ESM.docx]

***Gender dynamics in seed systems: female makeover or male takeover of specialized sweetpotato seed production, in Lake Zone Tanzania?***

*(Running title - Gender dynamics in specialized sweetpotato seed production)*

Margaret A. McEwan^1,2^, Moses S. Matui^2^, Sarah Mayanja^3^, Sam Namanda^4^, Kwame Ogero^5^

^1^Regional Office for Sub Saharan Africa, International Potato Center, P.O. Box 25171- 00603, Nairobi, Kenya. Email: [M.McEwan@cgiar.org](mailto:M.McEwan@cgiar.org)

^2^Wageningen University Department of Social Sciences, Wageningen University and Research, Hollandseweg 1, 6706 KN Wageningen, Netherlands. Email: [mosessila@gmail.com](mailto:mosessila@gmail.com)

^3^International Potato Center, P.O Box 22274, Kampala, Uganda. Email: [S.Mayanja@cgiar.org](mailto:S.Mayanja@cgiar.org)

^4^International Potato Center, P.O. Box 22274, Kampala, Uganda. Email: [S.Namanda@cgiar.org](mailto:S.Namanda@cgiar.org)

^5^International Potato Center, Tanzania. c/o Tanzania Agricultural Research Institute (TARI) –Ukiriguru, PO Box 1433, Mwanza, Tanzania. Email: [K.Ogero@cgiar.org](mailto:K.Ogero@cgiar.org)

ORCID

Margaret McEwan: [0000-0001-8510-0526](https://orcid.org/0000-0001-8510-0526)

Moses Matui: 000-0003-0750-4271

Sarah Mayanja: [0000-0002-9698-0036](https://orcid.org/0000-0002-9698-0036)

Sam Namanda: [0000-0001-7822-0626](https://orcid.org/0000-0001-7822-0626)

Kwame Ogero: 0000-0002-5141-6781

**Abstract**

Interest is growing for the development of inclusive seed production models. However, there is limited understanding of gender-based roles and constraints and how these might influence gender relations in seed production. Through a case study on sweetpotato seed production in Lake Zone Tanzania, this article examines men’s and women’s roles in seed production with the introduction of specialized seed practices and a commercial orientation. The study uses data from 17 field-based plot observations and eight sex disaggregated focus group discussions (FGDs) with 33 (51% women and 48% men) decentralized vine multipliers (DVMs). Participatory, gender-based analytical tools were used to obtain an in-depth understanding of gender dimensions and implications of new seed production practices, the resources required and access to those resources. Our findings show that men and women have complementary roles in specialized seed production, and that men increased their involvement in production and commercialization, especially when larger monetary inputs and transactions took place. Women gained new tangible (income) and intangible (knowledge) assets, which enhanced their community status. Women’s contributions to household income became more visible. In conclusion male-takeover did not take place. There were changes in the perceptions around sweetpotato production and gender relations. As women’s contributions to household income became more visible, they were able to negotiate with their husbands on access to key resources to maintain this household revenue stream. We discuss how the new knowledge and skills related to seed production enhanced women’s status in the community. These dynamics initiated changes in gender relations and challenged prevailing community perceptions on gender roles.

**Key words:** seed enterprises, gender-based roles, decentralized vine multipliers.
